# Light-Controlled Affinity Purification of Protein Complexes Exemplified by the Resting ZAP70 Interactome

**DOI:** 10.3389/fimmu.2019.00226

**Published:** 2019-02-26

**Authors:** Maximilian Hörner, Julian Eble, O. Sascha Yousefi, Jennifer Schwarz, Bettina Warscheid, Wilfried Weber, Wolfgang W. A. Schamel

**Affiliations:** ^1^Faculty of Biology, University of Freiburg, Freiburg, Germany; ^2^Signalling Research Centres BIOSS and CIBSS, University of Freiburg, Freiburg, Germany; ^3^Spemann Graduate School of Biology and Medicine, University of Freiburg, Freiburg, Germany; ^4^Centre for Chronic Immunodeficiency CCI, Medical Center, University of Freiburg, Freiburg, Germany

**Keywords:** optogenetics, affinity purification, phytochrome, ZAP70, protein-protein interaction, mass spectrometry

## Abstract

Multiprotein complexes control the behavior of cells, such as of lymphocytes of the immune system. Methods to affinity purify protein complexes and to determine their interactome by mass spectrometry are thus widely used. One drawback of these methods is the presence of false positives. In fact, the elution of the protein of interest (POI) is achieved by changing the biochemical properties of the buffer, so that unspecifically bound proteins (the false positives) may also elute. Here, we developed an optogenetics-derived and light-controlled affinity purification method based on the light-regulated reversible protein interaction between phytochrome B (PhyB) and its phytochrome interacting factor 6 (PIF6). We engineered a truncated variant of PIF6 comprising only 22 amino acids that can be genetically fused to the POI as an affinity tag. Thereby the POI can be purified with PhyB-functionalized resin material using 660 nm light for binding and washing, and 740 nm light for elution. Far-red light-induced elution is effective but very mild as the same buffer is used for the wash and elution. As proof-of-concept, we expressed PIF-tagged variants of the tyrosine kinase ZAP70 in ZAP70-deficient Jurkat T cells, purified ZAP70 and associating proteins using our light-controlled system, and identified the interaction partners by quantitative mass spectrometry. Using unstimulated T cells, we were able to detect the known interaction partners, and could filter out all other proteins.

## Introduction

Most, if not all, biochemical processes in cells, such as signal transduction, rely on protein-protein-interactions (1, 2). In the cells of the immune system for example, signalosomes change in their composition upon stimulation of cell surface receptors (3, 4). A well-studied example is the binding of the T cell antigen receptor (TCR) complex to the tyrosine kinase ZAP70 in resting T cells (5–8). Upon ligand binding to the TCR, ZAP70 gets activated by phosphorylation and detaches from the TCR (9) leading to the generation of new intracellular interactions and the remodeling of signalosomes (10). As those protein-protein-interactions control cell behavior, their investigation is of key interest in immunological and biological research.

Interaction partners of proteins are usually identified by purification of a protein of interest (POI) and analyzing the co-purified proteins by mass spectrometry (11, 12). Besides the use of POI-specific antibodies, one common approach is the purification of the POI via an affinity tag. There, the POI is expressed as a fusion protein with an affinity tag that binds specifically to a resin material. Then the cells expressing the fusion protein are lysed with a detergent, insoluble material is removed by centrifugation, and the lysate is added to the resin. Subsequently, the POI together with its interacting proteins binds to the resin. After washing, the POI together with its interaction partners can be eluted from the resin, e.g., by a change in the pH (FLAG tag, HA tag), ion concentration (Poly-Arg tag), temperature (protein G tag), or by addition of metal chelators (CBP tag) or molecules that compete with the binding of the affinity tag to the resin (Strep tag, SBP tag, Poly-His tag) (13, 14). Alternatively, the affinity tag is linked to the POI using a sequence that can be cleaved by a protease, such as Tobacco Etch Virus (TEV) protease (15). Hence, elution is achieved by adding the protease, cleaving the POI off the resin. Importantly, all the different approaches have in common that the changed biochemical environment in the elution step can also result in the release of unspecifically bound proteins from the resin material resulting in false positive hits in the following analysis. One approach to reduce the number of contaminants in the eluate is the sequential usage of two different affinity tags, named tandem affinity purification (TAP) (16). However, due to the thereby prolonged time needed for purification, more transient and weak interactors may be lost during this procedure.

In this study, we address this problem by developing an optogenetics-based purification approach allowing elution of tagged proteins by simply changing the wavelength of light with which the resin is illuminated. Thus, all biochemical parameters, such as the ones mentioned above, stay constant, minimizing the elution of proteins that were bound to the resin and not to the POI. We made use of the light-dependent protein-protein interaction between phytochrome B (PhyB) and its phytochrome interacting factor 6 (PIF6) both from *Arabidopsis thaliana* (17). Upon illumination with 660 nm red light PhyB switches to its Pfr conformational state (PhyB far-red absorbing state) in which it interacts with PIF6 with a nanomolar affinity (18). With 740 nm far-red light PhyB undergoes a conformational transition to the Pr state (PhyB red absorbing state) preventing binding to PIF6. This light-dependent protein-protein interaction was applied for several optogenetic applications (19), such as the control of protein or organelle localization (18, 20), signaling (21), nuclear transport of proteins (22), or gene expression (23).

Here, we make the red light-dependent interaction between PhyB and PIF6 applicable to the affinity purification of protein complexes. To this end, we identified a truncated variant of PIF6 comprising only 22 amino acids that reversibly interacts with PhyB and therefore can be used as an affinity tag for the POI. After characterization of the key parameters of our light-controlled affinity purification approach, we applied our method for the identification of interaction partners of ZAP70 in resting T cells by quantitative mass spectrometry.

## Results and Discussion

**Figure 1:**
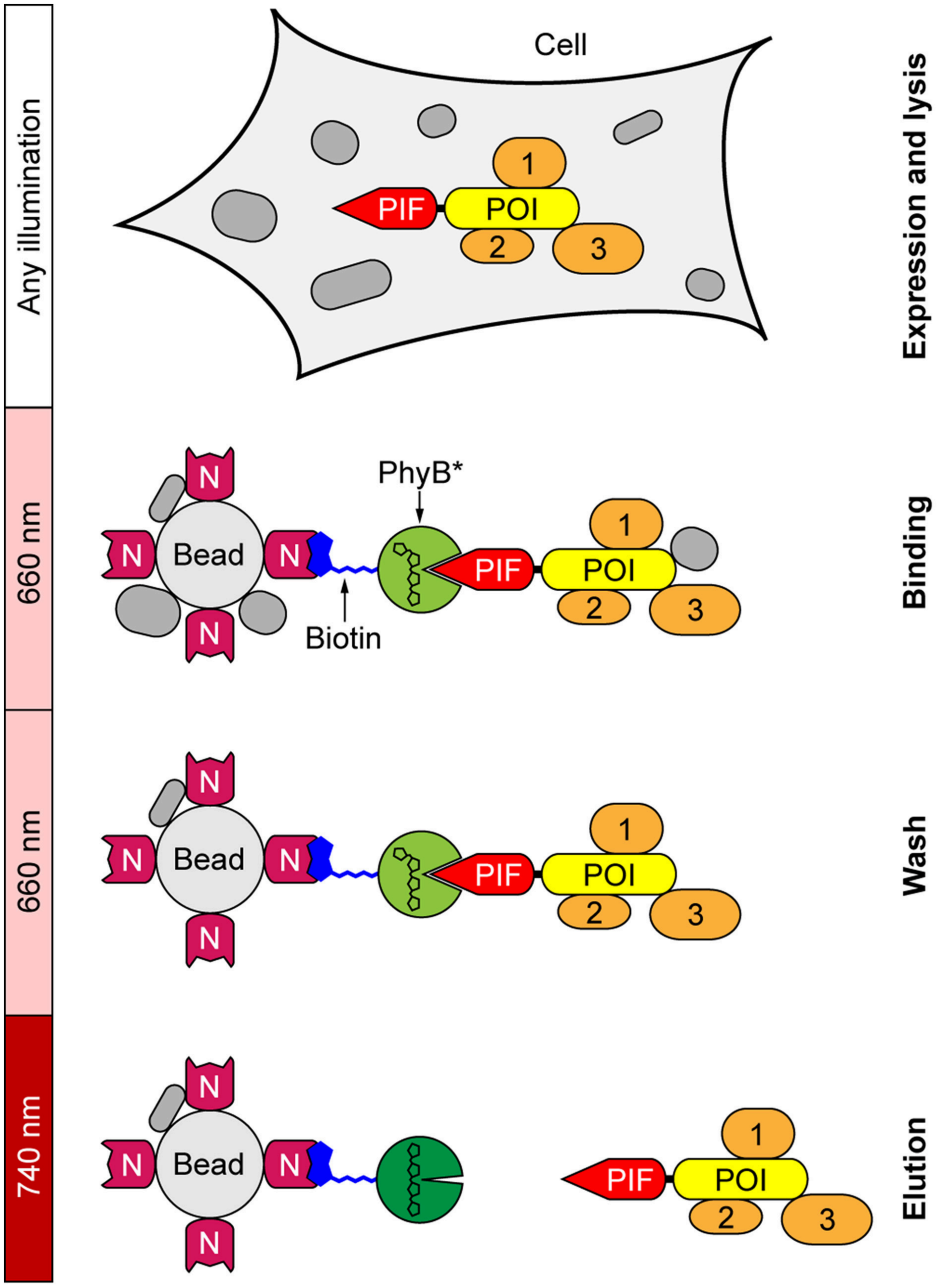
Light-controlled affinity purification of proteins. The protein of interest (POI) is expressed in the desired cells as a fusion protein with a truncated variant of phytochrome interacting factor 6 (PIF) serving as the affinity tag. Biotinylated phytochrome B (PhyB^*^) is immobilized on NeutrAvidin (N)-functionalized agarose beads. Following cell lysis, the POI is bound via its PIF tag to PhyB^*^ under 660 nm light. After washing to remove unspecifically bound proteins under continued 660 nm illumination, PIF-POI is eluted in washing buffer from PhyB^*^ beads by switching illumination to 740 nm light. Interaction partners (1–3) of the POI are co-purified.

In our new light-controlled affinity purification approach, a fusion protein between the POI and a truncated version of PIF6 is expressed in the desired cells (Figure 1). After cell lysis, the lysate is loaded under 660 nm light illumination onto agarose beads that have been functionalized with PhyB. Illumination with 660 nm light switches PhyB into the Pfr state, thus immobilizing the PIF-POI fusion protein and potential interaction partners to the PhyB beads. Afterwards the beads are washed under continued 660 nm illumination for removal of unspecific bound proteins. Finally, PIF-POI and its binding partners are eluted in the same buffer as used in the washing steps by simply changing illumination to 740 nm light, as light of this wavelength switches PhyB into the Pr state that terminates the interaction with PIF.

### PhyB^*^-Functionalized Resin Material

In order to functionalize agarose beads with PhyB, we used a biotinylated and hexahistidine-tagged variant of PhyB comprising amino acids 1-651 (24). This protein was produced together with the enzymes for the biosynthesis of the required chromophore phycocyanobilin (PCB) in *E. coli*. We purified this PhyB variant (designated as PhyB^*^) via immobilized metal ion affinity chromatography and quantified its biotinylation by binding of the protein to NeutrAvidin-functionalized agarose beads. Using an excess of beads, we observed that ~98% of PhyB^*^ was biotinylated (Supplementary Figure 1). To determine the PhyB^*^ binding capacity of the NeutrAvidin agarose beads, we incubated a fixed amount of beads with increasing amounts of PhyB^*^ and monitored a decrease in the ratio of beads-bound PhyB^*^ (Supplementary Figure 1). Saturation of the beads was reached with approximately 0.2 nmol PhyB^*^ per 1 μl beads. In the following, 1 μl NeutrAvidin beads were always incubated with ~0.27 nmol PhyB^*^ resulting in a coupling of ~0.24 nmol PhyB^*^ per 1 μl of beads (~89% coupling efficiency, designated as PhyB^*^ beads).

### Truncation of the PIF6 Tag

**Figure 2:**
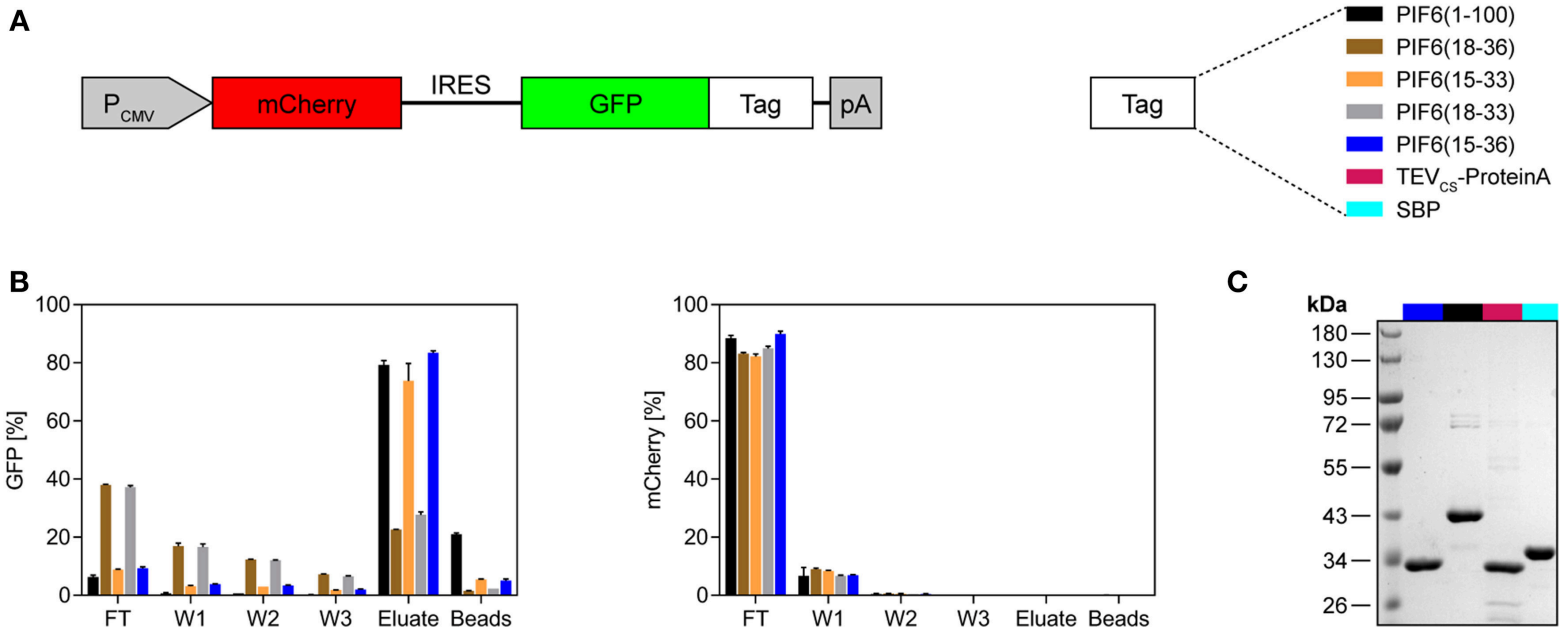
Evaluation of different truncated variants of PIF6 for the application as an affinity tag. **(A)** Design of the expression construct for testing of different affinity tags. GFP was fused to the depicted PIF6 variants or to the affinity tags TEV_CS_-ProteinA or SBP. The fusion proteins were expressed together with the untagged red fluorescent protein mCherry by a constitutive CMV promoter in human embryonic kidney cells (HEK-293T). IRES, internal ribosomal entry site; pA, poly(A) tail. **(B)** Monitoring of the light-controlled affinity purification process. Fluorescence of GFP and of the background control mCherry was measured using a plate reader and is shown as percentage normalized to the fluorescence of the initial cell lysate. For each replicate, 4 × 10^6^ cells were lysed in 500 μl lysis buffer and proteins were purified with 50 μl of PhyB^*^ beads. The proteins were eluted at 740 nm light for 30 min. FT, flow-through; W1-W3, wash 1-3. Data are means ± s.d. (*n* = 2). **(C)** Comparison of the purity of light-controlled affinity purification [PIF6(15-36) and PIF6(1-100)] with established purification methods (TEV_CS_-ProteinA and SBP). For each purification, 125 × 10^6^ cells were lysed with 500 μl of lysis buffer and purified with 100 μl of beads and four washing steps. Coomassie-stained SDS-PAGE gel of the eluates is shown.

It was shown that the N-terminal 100 amino acids of *A. thaliana* PIF6 [PIF6(1-100)] are sufficient for the reversible and light-dependent interaction with PhyB (17, 18). It is desirable to minimize the size of an affinity tag in order to disturb the fused POI as minimally as possible and to reduce undesired protein binding to the tag. Therefore, we aimed to further truncate PIF6(1-100) while maintaining its light-dependent interaction properties with PhyB. To this end, we performed a sequence alignment of different PIF variants from several plants to identify a region within the N-terminal 100 amino acids that is well-conserved and hence should constitute the core domain of PIF6 responsible for the light-dependent interaction with PhyB (Supplementary Figure 2). We found a conserved region between amino acids 15 and 36. Thus, we tested four different truncated PIF6 variants containing different amino acids within the 15–36 region and compared them to PIF6(1-100) (Figure 2A). To this end, we expressed the green fluorescent protein (GFP) fused to the selected PIF6 variants together with the untagged red fluorescent protein mCherry in human embryonic kidney (HEK)-293T cells. Subsequently, PIF-tagged GFP was purified from the cell lysates with the PhyB^*^-functionalized beads and spin columns. During the purification process, we monitored the fluorescence of mCherry as a background control and of GFP as our POI (Figure 2B). We observed that all tested PIF tags allowed a light-controlled purification of the fused GFP. Remarkably, PIF6(15-33) and PIF6(15-36) showed a similar efficiency compared to PIF6(1-100). The amount of mCherry in the eluates was at least 4,000-fold reduced compared to the lysates, demonstrating the specificity of our purification protocol. Next, we compared our light-controlled purification approach with established affinity chromatography methods. These were based on the streptavidin binding peptide [SBP tag (25)] or on the TEV mediated cleavage of the POI from protein A [TEV_CS_-ProteinA tag (26)]. We observed a similar recovery (70–80%, Supplementary Figure 3) and purity of the eluates (Figure 2C).

### Characterization of the Purification Process

**Figure 3:**
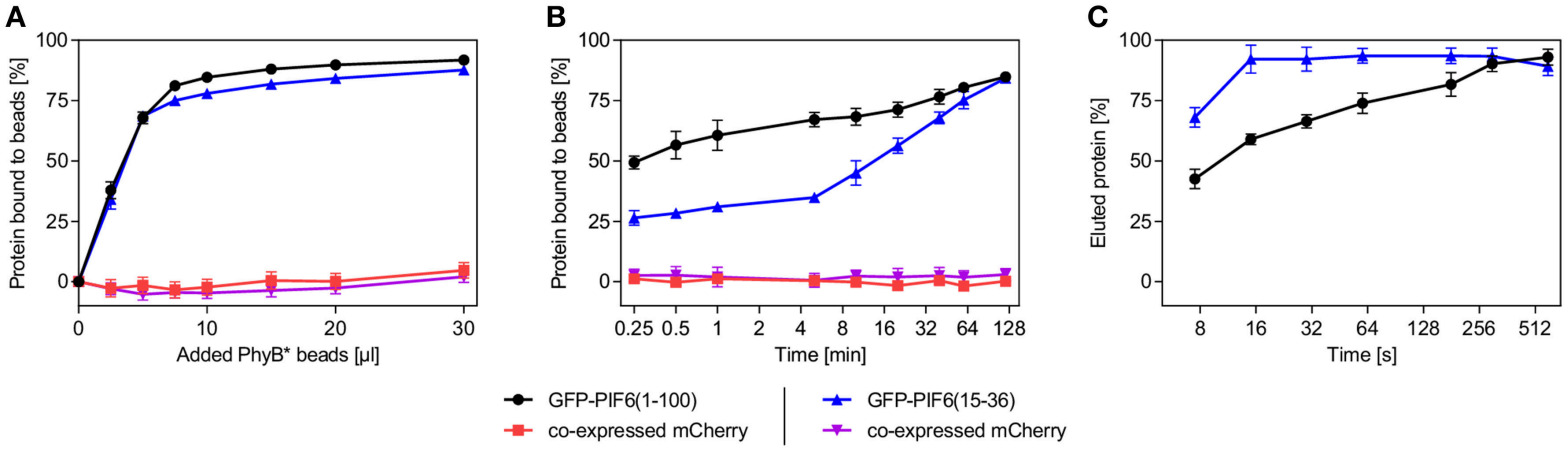
Characterization of the light-controlled affinity purification system. Lysates of HEK-293T cells containing mCherry and GFP-PIF6(1-100) or GFP-PIF6(15-36) as described in Figure 2A were used. **(A)** Binding efficiency and capacity of PhyB^*^ beads. Different amounts of PhyB^*^ beads were each incubated with 250 μl of cell lysate containing 1.5 nmol GFP-PIF6(1-100) or GFP-PIF6(15-36) for 2 h at 660 nm illumination. Afterwards, the ratio of bound protein was determined by measuring fluorescence of unbound GFP and mCherry. **(B)** Binding kinetics. Equal amounts of PhyB^*^ beads (20 μl) were incubated with 125 μl of cell lysate containing 0.75 nmol of GFP-PIF6(1-100) or GFP-PIF6(15-36) for the indicated time periods under 660 nm light. Subsequently, the ratio of bound protein was determined by measuring fluorescence of unbound GFP and mCherry. **(C)** Elution kinetics. Equal amounts of PhyB^*^ beads (40 μl) were incubated with 500 μl of cell lysate containing 3.0 nmol of GFP-PIF6(1-100) or GFP-PIF6(15-36) for 1 h at 660 nm illumination. After washing, the beads were incubated in 500 μl of wash buffer for the indicated times at 740 nm light for elution. The percentage of eluted protein was calculated by determining GFP fluorescence in the supernatant in comparison to the GFP amount on the beads before elution. All data are means ± s.d. (*n* = 3).

We characterized the purification process of GFP-PIF6(1-100) and GFP-PIF6(15-36) with PhyB^*^ beads in more detail. We determined that within a binding period of 2 h at 660 nm illumination both PIF-tagged proteins were binding equally well to the PhyB^*^ beads with a binding efficacy of ~85% (using an excess of PhyB^*^ beads, Figure 3A). When the PIF-tagged proteins were in excess compared to PhyB^*^, ~0.21 nmol of GFP-PIF6(1-100) or GFP-PIF6(15-36) were binding per 1 μl of the PhyB^*^ beads. We further observed that GFP-PIF6(1-100) was binding faster to the PhyB^*^ beads under 660 nm light than GFP-PIF6(15-36) with 50% bound protein within 0.25 min compared to 15 min, respectively (Figure 3B). In contrast, GFP-PIF6(1-100) eluted slower from the PhyB^*^ beads under 740 nm illumination than GFP-PIF6(15-36) with 80% released protein within 180 s compared to 16 s, respectively (Figure 3C).

### Generation of ZAP70-PIF6 Tag Cell Lines

**Figure 4:**
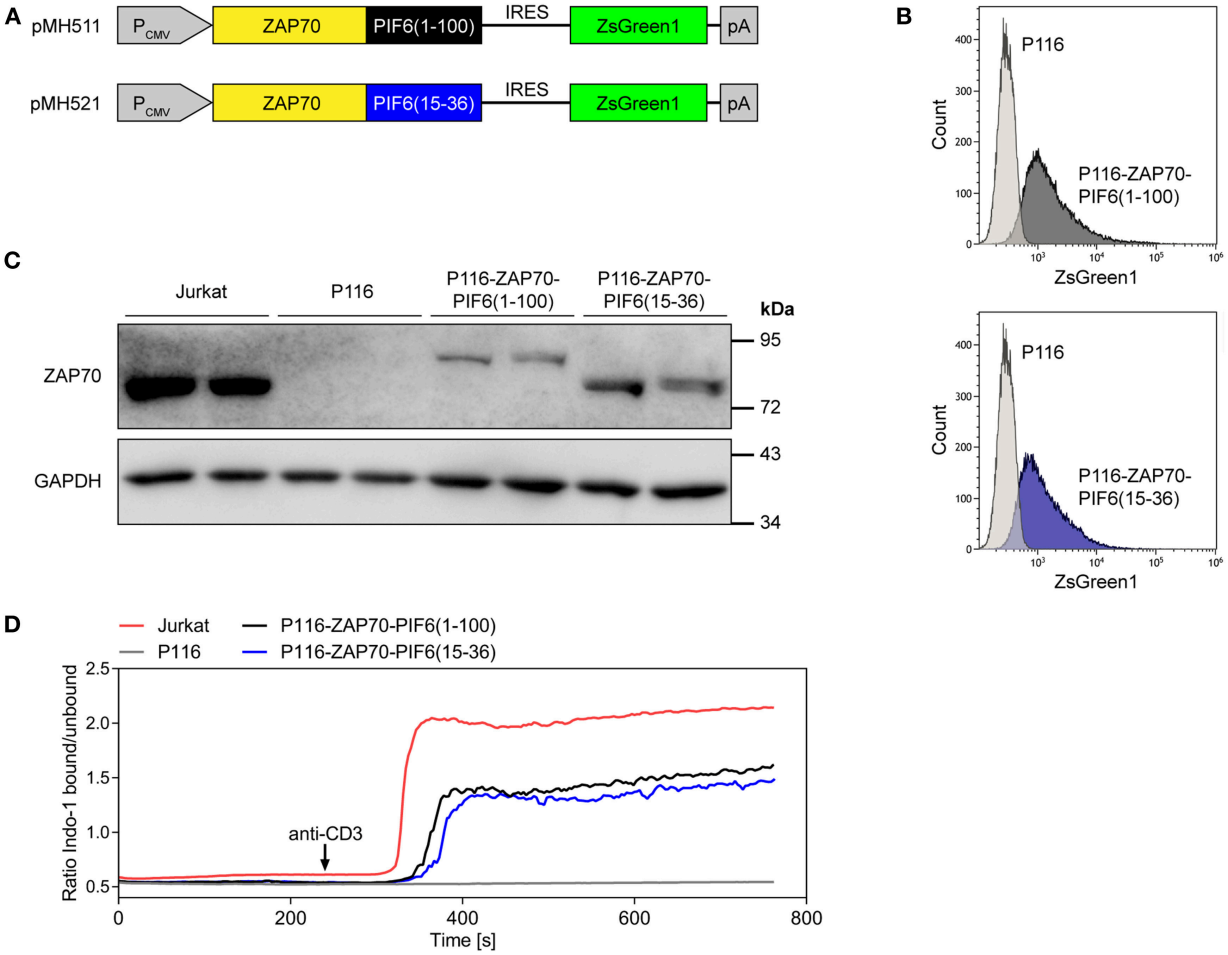
Generation and characterization of P116-based cell lines stably expressing ZAP70-PIF6(1-100) or ZAP70-PIF6(15-36). **(A)** Lentiviral vectors encoding the depicted constructs for expression of ZAP70-PIF6(1-100) or ZAP70-PIF6(15-36) and the fluorescent protein ZsGreen1 under control of the constitutive CMV promoter (P_CMV_). Transduced P116 cells were sorted based on ZsGreen1 fluorescence. **(B)** After culturing the two sorted cell lines generated in **(A)**, ZsGreen1 fluorescence of the cell lines in comparison to parental P116 cells was analyzed by flow cytometry. **(C)** Expression of ZAP70 in different cell lines. The cell lines indicated were analyzed for ZAP70 and GAPDH expression by SDS-PAGE and Western blotting. **(D)** Restoring anti-CD3 induced Ca^2+^-flux in P116 cells stably expressing ZAP70-PIF6(1-100) or ZAP70-PIF6(15-36). The Indo-1 fluorescence of Indo-1-stained cells was measured by flow cytometry, as a readout for the intracellular Ca^2+^ concentration. After 240 s of the recording the cells were stimulated with 1 μg ml^−1^ of an anti-CD3 antibody (indicated by the arrow).

We tested our light-controlled purification approach for the identification of interaction partners of the kinase ZAP70 by quantitative mass spectrometry. ZAP70 is a central kinase involved in TCR signaling, which interacts with the components of the TCR such as CD3δ, CD3ε, CD3γ, or CD247 (5, 6, 10). We transduced the ZAP70-deficient Jurkat cell line P116 (27) with constructs expressing PIF6(1-100)- or PIF6(15-36)-tagged human ZAP70 and the fluorescent protein ZsGreen1 (Figure 4A). Based on the fluorescence of the marker ZsGreen1, we sorted transduced cells with low ZsGreen1 expression levels using flow cytometry to avoid overexpression of our ZAP70 fusion proteins. As expected, most of the sorted cells stably expressed ZsGreen1 as shown by flow cytometry (Figure 4B). Expression and correct size of the fusion proteins were confirmed by SDS-PAGE and Western Blotting using an anti-ZAP70 antibody (Figure 4C). Both tagged ZAP70 proteins were expressed to lower levels than endogenous ZAP70 in the Jurkat cell line. ZAP70 is directly downstream of the TCR, transducing TCR signals into the cell, such as Ca^2+^ influx into the cytosol. Indeed, when stimulating the TCR with anti-CD3 antibodies we detected a Ca^2+^ response in Jurkat cells, but not in P116 cells (Figure 4D). Moreover, the restored anti-CD3 induced Ca^2+^ influx in our stable cell lines showed that the fusion of the PIF tags to ZAP70 did not impair the functionality of ZAP70 (Figure 4D). The reduced Ca^2+^-flux in comparison to Jurkat cells can likely be attributed to the reduced expression level of our PIF-ZAP70 fusion constructs in comparison to the endogenous ZAP70 level of Jurkat cells (Figures 4C,D).

### Identification of ZAP70 Interaction Partners by Mass Spectrometry

**Figure 5:**
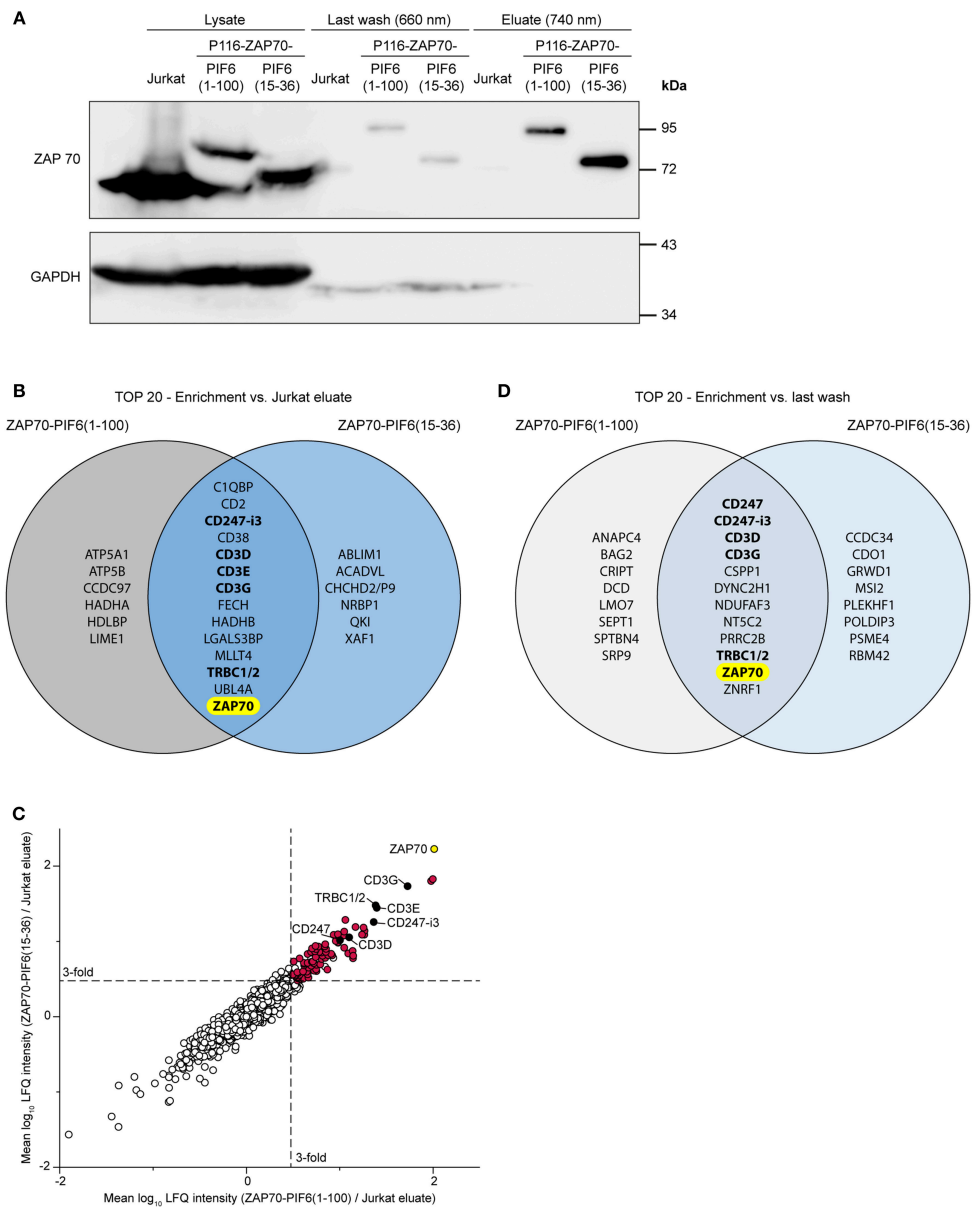
Analysis of light-controlled ZAP70-PIF purification by mass spectrometry. Jurkat cells and P116-derived cell lines stably expressing ZAP70-PIF6(1-100) or ZAP70-PIF6(15-36) were lysed and PIF-tagged proteins were light-controlled purified using PhyB^*^ beads (*n* = 3 biological replicates). All eluates and the flow-throughs of the last washing were analyzed by mass spectrometry. **(A)** Analysis of the cell lysates, the flow-throughs of the last washing step and the eluates by SDS-PAGE and Western blot against ZAP70 and GAPDH. **(B)** Comparison of the 20 best hits (*p* < 0.05, sorted on protein enrichment) enriched in the eluates of each ZAP70-PIF6 cell line vs. the eluates from the Jurkat cell line. Known interaction partners of ZAP70 are written in bold. **(C)** Enrichment of proteins identified in the ZAP70-PIF6(1-100) eluate vs. the Jurkat eluate (x-axis) plotted against the enrichment of proteins in the ZAP70-PIF6(15-36) eluate vs. the Jurkat eluate (y-axis). The enrichment for each protein is shown as the mean log_10_ ratio of the label-free quantification (LFQ) intensities (3 biological replicates) of the compared samples. Known interaction partners of ZAP70 identified in **(B)** are highlighted in black and are labeled. All significant hits (*p* < 0.05) in the upper right quadrant are highlighted in red. The POI ZAP70 is highlighted in yellow. Dashed lines represent a ratio cut-off corresponding to a 3-fold enrichment compared to the Jurkat eluate. **(D)** Comparison of the 20 best hits (*p* < 0.05, sorted on protein enrichment) enriched in the eluates of each ZAP70-PIF6 cell line vs. the flow-throughs of the last washing step. Known interaction partners of ZAP70 are written in bold. CHCHD2/P9, CHCHD2, CHCHD2P9; CD247-i3, CD247 isoform 3; TRBC1/2, TRBC1, TRBC2.

To test our new purification approach, we used both P116-derived stable cell lines in resting state, i.e., unstimulated. We purified PIF-tagged ZAP70 from these cell lines using our PhyB^*^ beads and analyzed the cell lysates, the flow-throughs of the last washing step and the eluates by Western blotting against ZAP70 and GAPDH (Figure 5A). As a negative control, we performed the same purification with Jurkat cells expressing non-tagged ZAP70. As expected, the eluates of the stable cell lines contained PIF-tagged ZAP70 of the correct molecular weight whereas the eluates of the Jurkat cells did not contain detectable amounts of ZAP70. The eluates of all purified cells showed a massive reduction in the GAPDH level compared to the cell lysates. This experiment demonstrates that we were able to specifically purify our POI, in this case ZAP70, using the new light-controlled affinity purification approach.

To validate whether we are able to identify interaction partners of ZAP70 by our light-controlled purification approach and whether we can eliminate false positives, we used the unstimulated P116 cells expressing PIF6(1-100)- or PIF6(15-36)-tagged human ZAP70. The usage of unstimulated cells has the following advantage. ZAP70 was shown to interact with the TCR in resting cells (7) thus this interaction serves as our positive control. Importantly, this ZAP70 is not phosphorylated on tyrosines (7). Since these tyrosines have to be phosphorylated to serve as interactions sites for further signaling proteins (8), we do not expect any other interaction partners besides the TCR. Thus, we can assume that other proteins detected by mass spectrometry are false positives.

Next, we analyzed the eluates after 740 nm illumination and the flow-throughs of the last washing step of the above described unstimulated samples by mass spectrometry (3 biological replicates each, Supplementary Table 1). We first looked for proteins that were significantly enriched in the eluates derived from each ZAP70-PIF6 cell line compared to the eluates derived from the Jurkat cell line. We observed that for both ZAP70-PIF6 cell lines the 20 top hits (*p* < 0.05, sorted on protein enrichment) contained besides ZAP70 as the POI, the proteins CD247-i3 (i3, isoform 3), CD3D, CD3E, CD3G, and TRBC1/2 (Figure 5B). The different CD3 proteins, CD247 (also called CD3ζ) as well as TRBC1/2 (also called TCRβ chains) are all subunits of the TCR (28) which is known to associate with ZAP70 (5, 6, 29). As stated above, ZAP70 associates with the TCR in unstimulated primary T cells (7) and most likely also in unstimulated Jurkat cells although to lower levels (30, 31). Since ZAP70 that is associated with the resting TCR is not phosphorylated (7) and thus might not have other binding partners, we conclude that the other proteins might be false positives. The two PIF6 tags showed a similar performance as shown by the high overlap of the 20 best hits (14 out of 20) and as visualized by the clustering of the proteins along the diagonal when plotting the enriched proteins against each other (Figure 5C, Supplementary Table 2). Together, this indicates that both PIF6(1-100) and PIF6(15-36) are suitable affinity tags that allow purification, and identification of a POI together with its interaction partners.

As our light-controlled purification approach is characterized by the very mild elution condition (740 nm light), we next asked whether we could use the flow-throughs of the last washing step performed under 660 nm illumination as background control instead of the eluates from the Jurkat cell line (expressing untagged ZAP70). Indeed, using this procedure we could identify a similar enrichment of ZAP70 for both PIF6 tags and the known interaction partners CD247, CD247-i3, CD3D, CD3G, and TRBC1/2 within the 20 best hits (*p* < 0.05, sorted on protein enrichment, Figure 5D). Therefore, the flow-throughs of the last washing step offer an alternative as control to filter out unspecific and false positive proteins.

**Figure 6:**
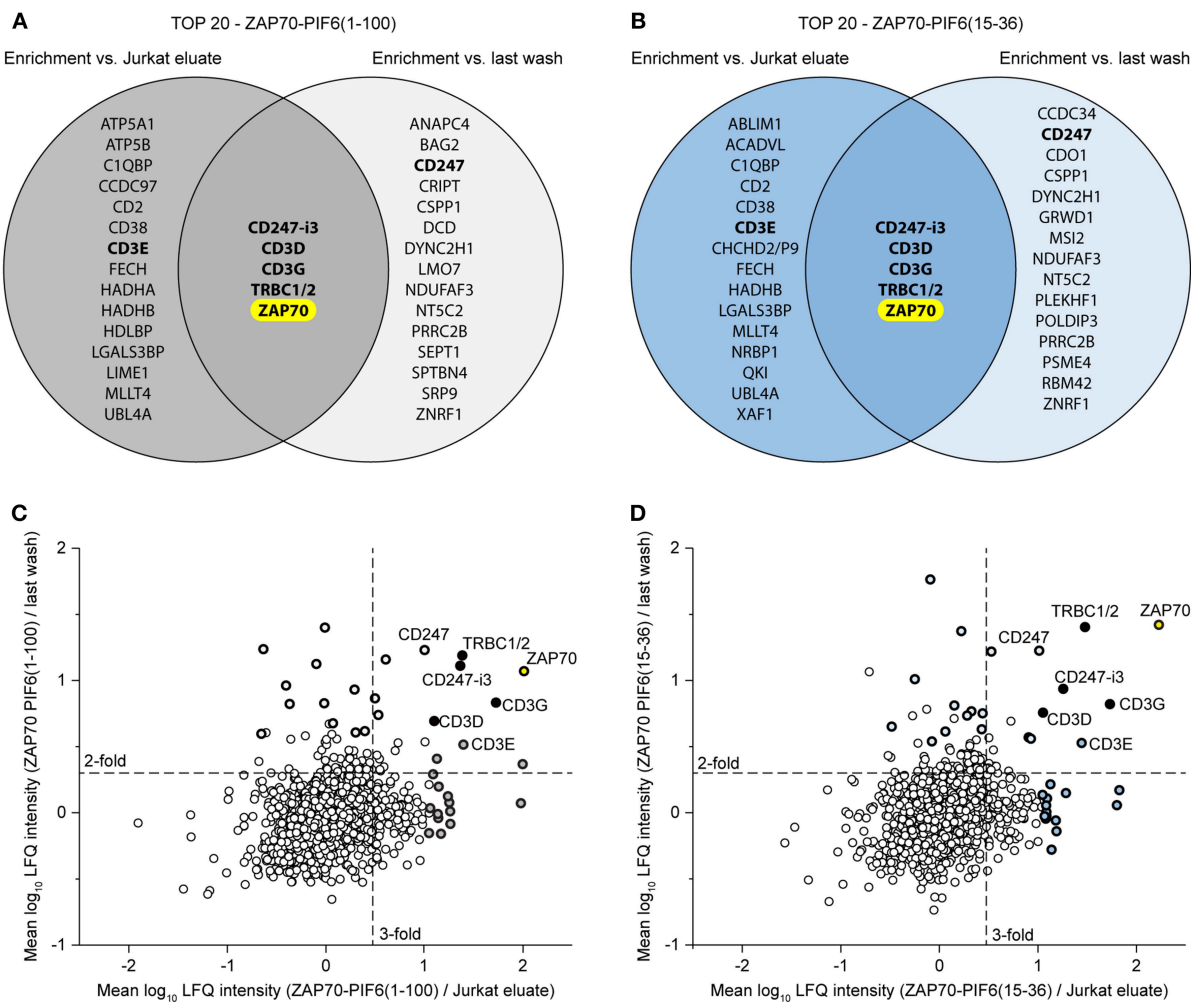
Usage of two controls to confine the data to the most relevant interaction partners of the POI ZAP70. **(A,B)** Comparison of the 20 best hits (*p* < 0.05, sorted on protein enrichment) enriched in the eluates of the ZAP70-PIF6(1-100) **(A)**/ZAP70-PIF6(15-36) **(B)** cell line vs. the eluates from the Jurkat cell line or the flow-throughs of the last washing step. Known interaction partners of ZAP70 are written in bold. **(C,D)** Enrichment of proteins identified in the ZAP70-PIF6(1-100) **(C)**/ZAP70-PIF6(15-36) **(D)** eluates vs. the Jurkat eluates (x-axis) plotted against the enrichment of proteins in the ZAP70-PIF6(1-100) **(C)**/ZAP70-PIF6(15-36) **(D)** eluates vs. the corresponding flow-throughs of the last washing step (y-axis). The enrichment for each protein is shown as the mean log_10_ ratio of the label-free quantification (LFQ) intensities (3 biological replicates) of the compared samples. Known double-positive interaction partners of ZAP70 identified within the TOP 20 hits in **(A)**/**(B)** are highlighted in black and the names are indicated; the remaining hits of the TOP 20 are highlighted as bold black circles filled with the color corresponding to the group color used in the VENN diagram in **(A)**/**(B)**. The POI ZAP70 is highlighted in yellow. Dashed lines represent a ratio cut-off corresponding to a 3-fold or 2-fold enrichment. CHCHD2/P9, CHCHD2, CHCHD2P9; CD247-i3, CD247 isoform 3; TRBC1/2, TRBC1, TRBC2.

Finally, we asked whether we could combine the Jurkat eluates and the flow-throughs of the last washing step to confine the interaction partner analysis to the most relevant hits. To this end, we compared for each PIF6 tag separately the 20 best hits (*p* < 0.05, sorted on protein enrichment) enriched in the eluates compared to the Jurkat eluates and to the flow-throughs of the last washing step (Figure 6, Supplementary Tables 3, 4). Identical for both PIF6 tags only ZAP70 and the known interaction partners CD247-i3, CD3D, CD3G, TRBC1/2 were overlapping. This suggests that the other proteins were indeed false positives that we were able to filter out. Of note, CD3E was classified as a false positive although it is a known component of the TCR and thus an interaction partner of ZAP70. Together our data suggest that the combined usage of the Jurkat eluates and the flow-throughs of the last washing step as controls can be used to confine the identified proteins to true interaction partners eliminating false positives.

## Conclusion

In this study we established an optogenetics-based light-controlled affinity purification method making use of the plant PhyB/PIF system. The POI-PIF6 fusion protein is recombinantly produced and bound to a PhyB^*^ resin. It is eluted after affinity purification by changing the wavelength of light from 660 to 740 nm. Compared to other affinity purification methods (see introduction), in which the biochemical or biophysical properties of the elution buffer are changed compared to the washing buffers, light in the red spectrum at the used low intensities is a mild elution condition. Thus, we can reduce the identification of false positives, especially if one uses two controls, namely the flow-through of the last washing step and control cells expressing the untagged POI.

The fast kinetics of the binding and dissociation of the PhyB/PIF system enable to conduct the affinity purification within a short time window, which is beneficial for the investigation of transient and weak interactions. Finally, we reduced the size of the PhyB-interacting fragment of PIF6 from 100 amino acids [PIF6(1-100)] to 22 amino acids [PIF6(15-36)]. The size reduction of the PIF6 tag increases its suitability as affinity tag by decreasing the probability to interfere with the function of the POI and to interact with other proteins. Furthermore, the 22 amino acids PIF6 fragment might also allow novel optogenetic applications using the PhyB/PIF system (19, 32).

## Materials and Methods

### Construction of Plasmids

The design and construction of the plasmids generated in this study are described in Supplementary Table 5 and the oligonucleotides used for this purpose in Supplementary Table 6. DNA-fragments were amplified by polymerase chain reaction and assembled by an isothermal, enzymatic DNA assembly reaction as described previously (Gibson cloning) (33).

### Production of PhyB^*^ and Protein Quantification

PhyB^*^ encoded by plasmid pMH17 was expressed in *E. coli* together with the biosynthesis genes for phycocyanobilin (PCB) encoded by plasmid p171 and purified by immobilized metal ion affinity chromatography (IMAC) as described previously (24). The fluorescent protein GFP-PIF6(1-100)-His_6_ encoded by plasmid pHB111 was used as fluorescence standard and was produced in *E. coli* and purified by IMAC as described previously (34). The protein concentrations of the purified proteins were determined by Bradford assay (Bio-Rad, Hercules, CA, cat. no. 500-0006) using bovine serum albumin (BSA, Sigma-Aldrich, St. Louis, MO, cat. no. 05479) as standard.

The fluorescent proteins GFP and mCherry were quantified by measuring their fluorescence (GFP: excitation: 488 nm, emission: 522 nm; mCherry: excitation: 588 nm, emission: 620 nm) with an Infinite M200 Pro microplate reader (Tecan, Männedorf, Switzerland). The concentration of all GFP-containing proteins expressed in HEK-293T cells was calculated based on the fluorescence using purified GFP-PIF6(1-100)-His_6_ in wash buffer [20 mM Tris/HCl, 137 mM NaCl, 2 mM EDTA, 10% (v/v) glycerol, pH 7.4] as standard. In Supplementary Figure 1, the concentration of unbound PhyB^*^ was determined by measuring PhyB^*^ fluorescence (excitation: 620 nm, emission: 680 nm) after 660 nm illumination (10 min, 20 μmol m^−2^ s^−1^) with an Infinite M200 Pro microplate reader.

### Maintenance of Mammalian Cells

HEK-293T cells were maintained in DMEM complete medium [DMEM (PAN Biotech, Aidenbach, Germany, cat. no. P04-03550), 10% (v/v) fetal calf serum (FCS, PAN Biotech, cat. no. P30-3602), 100 U ml^−1^ penicillin, 100 μg ml^−1^ streptomycin]. Jurkat, P116 (27) and transduced P116 cells were cultivated in RPMI complete medium [RPMI 1640 (Thermo Fisher Scientific, Waltham, MA, cat. no. 31870-025), 10% (v/v) FCS (PeproTech, Hamburg, Germany, cat. no. 200-02), 2 mM L-glutamine, 100 U ml^−1^ penicillin, 100 μg ml^−1^ streptomycin, 10 mM HEPES (Thermo Fisher Scientific, cat. no. 15630-056)]. All cells were incubated in a humidified atmosphere at 37°C, 5% CO_2_.

### Expression and Harvesting of GFP Fused to Different Affinity Tags

All constructs encoding untagged mCherry and GFP fused to different affinity tags (Figure 2A, Supplementary Table 5) were expressed in HEK-293T cells. To this aim, 6 × 10^6^ HEK-293T cells were seeded per 15 cm culture dish and transfected 24 h later with the corresponding plasmid. Per 15 cm dish, 60 μg plasmid DNA, and 200 μg PEI (linear, MW: 25 kDa, Polysciences, Warrington, PA, cat. no. 23966-2) were mixed in 3 ml OptiMEM (Thermo Fisher Scientific, cat. no. 22600-134), incubated for 15 min at RT and added dropwise to the cells. After incubation for 5 h, the medium was exchanged and proteins were expressed for 48 h. Before harvesting, the cells were washed once with DBPS containing Ca^2+^ and Mg^2+^ (PAN Biotech, cat. no. P04-35500) and cells were lysed–if not stated otherwise–with 3 ml of lysis buffer [20 mM Tris/HCl, 137 mM NaCl, 2 mM EDTA, 10% (v/v) glycerol, pH 7.4, protease inhibitor cocktail (Sigma-Aldrich, cat. no. SRE0055), 0.5% (v/v) Brij97 (Sigma-Aldrich, cat. no. 431281-100ML)] per 15 cm culture dish for 20 min on ice. After clarifying the lysate by centrifugation at 16,500 × g for 15 min, the supernatant was either immediately further processed or shock-frozen in liquid nitrogen and stored at −80°C. The average yield of GFP-PIF6(1-100) was ~720 μg of purified protein per 15 cm dish.

### Light-Controlled Affinity Purification

NeutrAvidin agarose beads (Thermo Fisher Scientific, cat. no. 29202) were washed once with Ni-Elution buffer (50 mM NaH_2_PO_4_, 300 mM NaCl, 250 mM imidazole, pH 8.0) and incubated afterwards with purified PhyB^*^ in Ni-Elution buffer supplemented with 1 mM 2-mercaptoethanol (final PhyB^*^ concentration: ~2 mg ml^−1^) for 2 h at 4°C under slight agitation. If not otherwise stated, 1 μl NeutrAvidin beads were incubated with 0.27 nmol PhyB^*^. After washing the beads twice with wash buffer, the beads were resuspended in lysis buffer and the indicated amounts of beads were transferred into a spin column (Thermo Fisher Scientific, cat. no. 69725). Subsequently, the cell lysate was added to the beads and the closed spin column was incubated on a rotator for 60 min under 660 nm illumination (10 μmol m^−2^ s^−1^) at 4°C. All following steps were performed under green safe light at 4°C. After incubation, the beads were washed by centrifugation of the spin column at 150 × g for 30 s, addition of 500 μl of wash buffer and incubation of the closed spin column for 10 min on a rotator under 660 nm illumination. This washing step was repeated for the indicated number of times (3 x if not indicated otherwise). Afterwards, the column was centrifuged before 500 μl of wash buffer were added and the column was incubated under 740 nm light (70 μmol m^−2^ s^−1^) on a rotator for 10 min. Finally, the eluted proteins were collected by centrifugation of the spin column.

### Affinity Purification of GFP With SBP Tag

The indicated number of HEK-293T cells transfected with plasmid pMH501 (as described above) were lysed 48 h after transfection with 500 μl of lysis buffer. All following steps were performed at 4°C. The soluble fraction of the cell lysate was incubated with the indicated amount of prewashed streptavidin Sepharose beads (GE Healthcare, Freiburg, Germany, cat. no. 17-5113-01) within a closed spin column on a rotator at 4°C for 2 h. Afterwards, the beads were washed by centrifugation (150 × g, 30 s) of the column, addition of 500 μl of wash buffer and incubation of the closed spin column for 10 min on a rotator. After repeating this washing step for three times, the column was centrifuged and 500 μl of elution buffer (wash buffer supplemented with 2 mM biotin) was added and the closed spin column was incubated on a rotator at 4°C for 20 min. Finally, the eluted proteins were collected by centrifugation.

### Affinity Purification of GFP With TEV_CS_-ProteinA Tag

The indicated number of HEK-293T cells transfected with plasmid pMH502 (as described above) were lysed 48 h after transfection with 500 μl of lysis buffer. All following steps were performed at 4°C. The soluble fraction of the lysate was incubated with the indicated amount of prewashed IgG Sepharose beads (35) within a closed spin column (Mobicol Classic with 35 μm pore size filter, MoBiTec, Göttingen, Germany, cat. no. M1003 and M513515) on a rotator at 4°C overnight. Afterwards, the beads were washed by centrifugation (150 × g, 30 s) of the column, addition of 500 μl of wash buffer and incubation of the closed spin column for 10 min on a rotator. After repeating this washing step three times, the columns were centrifuged and the beads were resuspended in 500 μl of wash buffer containing TEV protease (1 U TEV per 1 μl of beads, Thermo Fisher Scientific, cat. no. 12575015). After incubation on a shaker at 16°C for 3 h, prewashed Ni-NTA agarose beads (1 μl Ni-NTA beads per 2 μl of IgG beads) were added to remove the His_6_-tagged TEV protease and the sample was incubated for further 2 h. Finally, the eluted proteins were collected by centrifugation.

### Generation of Stable Cell Lines

The P116-based cell lines stably expressing ZAP70-PIF6(1-100) or ZAP70-PIF6(15-36) were generated by transduction with lentiviral particles. For lentiviral particle production, 8 × 10^6^ HEK293-T cells were seeded in DMEM lenti [Advanced DMEM (Thermo Fisher Scientific, cat. no. 12491015), 2% (v/v) FCS, 100 U ml^−1^ penicillin, and 100 μg ml^−1^ streptomycin, 10 μM cholesterol, 10 μM egg lecithin (Serva Electrophoresis, Heidelberg, Germany, cat. no. 27608), 1 x chemical defined lipid concentrate (Thermo Fisher Scientific, cat. no. 11905031)] per 15 cm dish. On the next day, the cells were transfected as described above with the plasmids pMH511 or pMH521, pLTR-G (36), and pCD/NL-BH^*^ΔΔΔ (37) in a mass ratio of 2:1:1 and the medium was replaced after 5 h with fresh DMEM lenti. After 48 h, the supernatant containing the lentiviral particle was harvested and filtered through a 0.45 μm filter. For transduction, P116 cells at a density of 3 × 10^5^ cells ml^−1^ were diluted 1:2 with the filtered lentiviral particle-containing supernatant. After 96 h, cells with low expression of the fluorescent protein ZsGreen1 were sorted using an S3e Cell Sorter (Bio-Rad).

### Lysis of T Cells

For Western blot analysis (Figure 4C) and for each mass spectrometry measurement, 1 × 10^6^ or 250 × 10^6^ cells were resuspended in 50 or 500 μl of lysis buffer supplemented with 1 mM phenylmethylsulfonyl fluoride, 5 mM iodoacetamide, 0.5 mM sodium orthovanadate, and 1 mM NaF, respectively. After incubation for 15 min on ice the lysate was clarified by centrifugation for 15 min at 16,500 × g. The supernatant was either mixed with SDS loading buffer for Western Blot analysis or used for light-controlled affinity purification (200 μl of PhyB^*^ beads, four washing steps) and subsequent mass spectrometry analysis.

### Western Blots

For Western blots, proteins were transferred after SDS-PAGE onto PVDF membranes and the membranes were blocked with TBS-T [TBS (50 mM Tris/HCl, 150 mM NaCl, pH 7.4) with 0.05% (v/v) Tween-20] containing 5% (w/v) milk powder for 1 h at RT. Afterwards, the membranes were incubated with the primary antibody (anti-ZAP70, dilution: 1:500, Santa Cruz Biotechnology, Dallas, TX, cat no. sc-30674 or anti-GAPDH, dilution: 1:100,000, Cell Signaling, Danvers, MA, cat. no. 5174) diluted in TBS-T supplemented with 3% (w/v) BSA and 0.02% (w/v) sodium azide at 4°C overnight. After washing the membrane three times with TBS-T, they were incubated with the secondary antibody (ZAP70: rabbit anti-goat IgG-HRP, dilution: 1:10,000, Thermo Fisher Scientific, cat. no. 31402 or GAPDH: goat anti-rabbit IgG-HRP, dilution: 1:10,000, Thermo Fisher Scientific, cat. no. 31460) diluted in TBS-T supplemented with 3% (w/v) BSA and 3% (w/v) milk powder for 1 h at RT. Following three washing steps with TBS-T, chemiluminescence was detected using ECL substrate and an ImageQuant LAS-4000 mini system (GE Healthcare).

### Ca^2+^-Flux Measurements

For measuring of Ca^2+^-flux, 5 × 10^6^ cells were washed with PBS and incubated in 1 ml starvation medium (RPMI complete containing 1% (v/v) FCS) containing 4 μM Indo-1 (Thermo Fisher Scientific, cat. no. I1223) and 0.1% (v/v) Pluronic F-127 (Thermo Fisher Scientific, cat. no. P3000MP) for 1 h at 37°C, 5% CO_2_. Afterwards, the cells were pelleted by centrifugation, resuspended in 500 μl of starvation medium and kept on ice in the dark until the measurement. Analyses of Ca^2+^-bound and unbound Indo-1 was performed with a customized MACSQuant Analyzer (Miltenyi Biotec, Bergisch Gladbach, Germany) using a 355 nm laser and 405/20 nm and 530/30 nm emission filters, respectively. Cells were stimulated with 1 μg ml^−1^ anti-CD3 antibody (UCHT1, Dr. Beverly, UK).

### Mass Spectrometry

The proteins present within 400 μl of the sample (either eluate or flow-through of the last washing step from the light-controlled affinity purification) were precipitated by addition of 1.6 ml of ice-cold acetone and incubation at −20°C overnight. Afterwards, the precipitated proteins were pelleted by centrifugation at 10,000 × g for 10 min at 4°C and the air-dried pellet was resolubilized in 10 μl of buffer (60% (v/v) methanol in 50 mM NH_4_HCO_3_). The disulfide bonds were then reduced with 5 mM tris(2-carboxyethyl)phosphine (TCEP) for 30 min at 65°C, and afterwards alkylated with 50 mM 2-chloroacetamide for 30 min at RT. The reaction was quenched by addition of 25 mM dithiothreitol (DTT), diluted with 25 μl buffer and the proteins were digested with 1 μg trypsin (Promega, Mannheim Germany, cat. no. V5111) at 42°C for 4 h. The samples were dried in a vacuum concentrator and resuspended in 15 μl of 0.1% (v/v) trifluoroacetic acid (TFA) prior to mass spectrometry (MS) analysis.

Liquid chromatography-tandem mass spectrometry (LC-MS/MS) analysis was performed with an UltiMate 3000 RLSCnano HPLC System (Thermo Fisher Scientific) coupled online to a Q Exactive Plus instrument (Thermo Fisher Scientific) essentially as described previously (38). Samples were washed on a C18 pre-column (Ø 0.3 × 5 mm; PepMap, Thermo Fisher Scientific) using 0.1% (v/v) TFA for 15 min, which was thereafter switched in line with the analytical column (Acclaim PepMap (ID: 75 μm × 250 mm, 2 μm, 100 Å, Dionex LC Packings/Thermo Fisher Scientific), equilibrated in 96% solvent A [0.1% (v/v) formic acid (FA)] and 4% solvent B (0.1% (v/v) FA, 86% (v/v) CH_3_CN). A gradient of 100 min (4–40% solvent B in 95 min and 40–95% solvent B in 5 min) at a flow rate of 0.250 μl min^−1^ was applied to separate the peptide mixture. The column was washed for 5 min with 95% solvent B, before re-equilibration for 15 min. The Q Exactive Plus acquired mass spectra from m/z 375 to 1,700, with a resolution of 70,000 at m/z 200 [parameters: automatic gain control (AGC), 3 × 10^6^ ions; maximum fill time, 60 ms]. The instrument operated in the data-dependent mode, using a TOP 15 method for the isolation of precursor ions (parameters: AGC target, 1 × 10^5^ ions; fill time, 120 ms; isolation window, 3 m/z; normalized collision energy, 28; underfill ratio, 1.2%; dynamic exclusion, 45 s).

### Mass Spectrometry Data Analysis

For mass spectrometry data analysis, MaxQuant version 1.5.5.1 with its integrated search engine Andromeda was used to search peak lists against the Uniprot proteome set *H. sapiens* (database 19/09/2018, 95109 entries) (39, 40). The search was conducted with default parameters (e.g., FDR 1% on protein and peptide level; precursor mass tolerance 20 ppm for the first search, 4.5 ppm for the main search), except the following adjustments: trypsin was selected as proteolytic enzyme and up to three missed cleavages were allowed. As variable modifications oxidation on methionine and acetylation on protein N-termini were selected. Cysteine carbamidomethylation was set as fixed modification. Protein identification required at least one unique peptide. Label-free quantification (LFQ) (41) was enabled, the LFQ minimal ratio count was set to 2 and fast LFQ was disabled. “Match between runs” was enabled with default parameters.

The LFQ intensities from the proteingroups.txt output file of MaxQuant were loaded into Perseus version 1.5.5.3 (42). Entries from contaminants, reverse hits and hits only identified by modified peptides were discarded. LFQ intensities were log_10_-transformed and proteingroups with 3 out of 3 valid values (3 independent biological replicates) in the ZAP70-PIF pulldown samples were used for further data analysis. Subsequently, for missing values of the control and wash samples random numbers were imputed from normal distribution (width of the distribution 0.5, down shift 1.8) to simulate values below the detection limit. To identify significantly enriched proteins, a right-sided, two-sample *t*-test was performed. Results were imported into Origin Pro 2017 (OriginLab, Northampton, MA) and visualized as scatter plots.

## Data Availability

The mass spectrometry proteomics data have been deposited to the ProteomeXchange Consortium via the PRIDE (43) partner repository with the dataset identifier PXD012156. All other raw data supporting the conclusions of this manuscript will be made available by the authors, without undue reservation, to any qualified researcher.

## Author Contributions

MH and JE planned and performed all experiments to set up the new purification protocol, and MH, OY, and JE the ones involving the P116 T cells. JS performed interactome studies by LC-MS followed by quantitative data analysis. MH, OY, BW, WW, and WS were involved in the design, supervision, and analysis of all experiments. MH, JE, and WS wrote the manuscript with input from all authors.

### Conflict of Interest Statement

The authors declare that the research was conducted in the absence of any commercial or financial relationships that could be construed as a potential conflict of interest.
