# Supplementary material for: Light-Controlled Affinity Purification of Protein Complexes Exemplified by the Resting ZAP70 Interactome
Source: Front Immunol. 2019 Feb 26;10:226. doi: 10.3389/fimmu.2019.00226 (PMC6399385; doi:10.3389/fimmu.2019.00226)
Supplement: Supplementary file 5 [file Data_Sheet_1.pdf]

Supplementary Material

Light-controlled affinity purification of protein complexes exemplified by the resting ZAP70 interactome

Maximilian Hörner, Julian Eble, O. Sascha Yousefi, Jennifer Schwarz, Bettina Warscheid, Wilfried Weber, Wolfgang W. A. Schamel

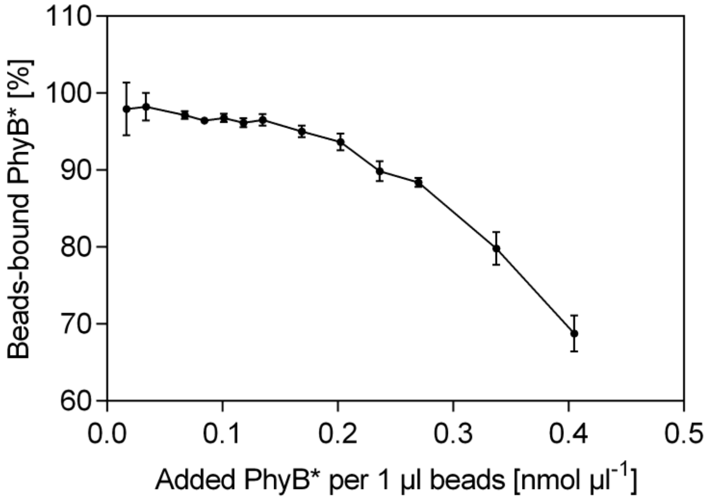

Supplementary Figure 1 | Characterization of PhyB* biotinylation and of the PhyB*-binding capacity of the NeutrAvidin agarose beads. Different amounts of PhyB* were incubated with 10 µl of NeutrAvidin beads for 1 h. Afterwards, the beads were pelleted by centrifugation and the fluorescence of unbound PhyB* in the supernatant was measured. Comparing this value with the PhyB* fluorescence before the addition of the beads allowed to calculate the percentage of beads-bound PhyB*. All data are means ± s.d. (n = 3).

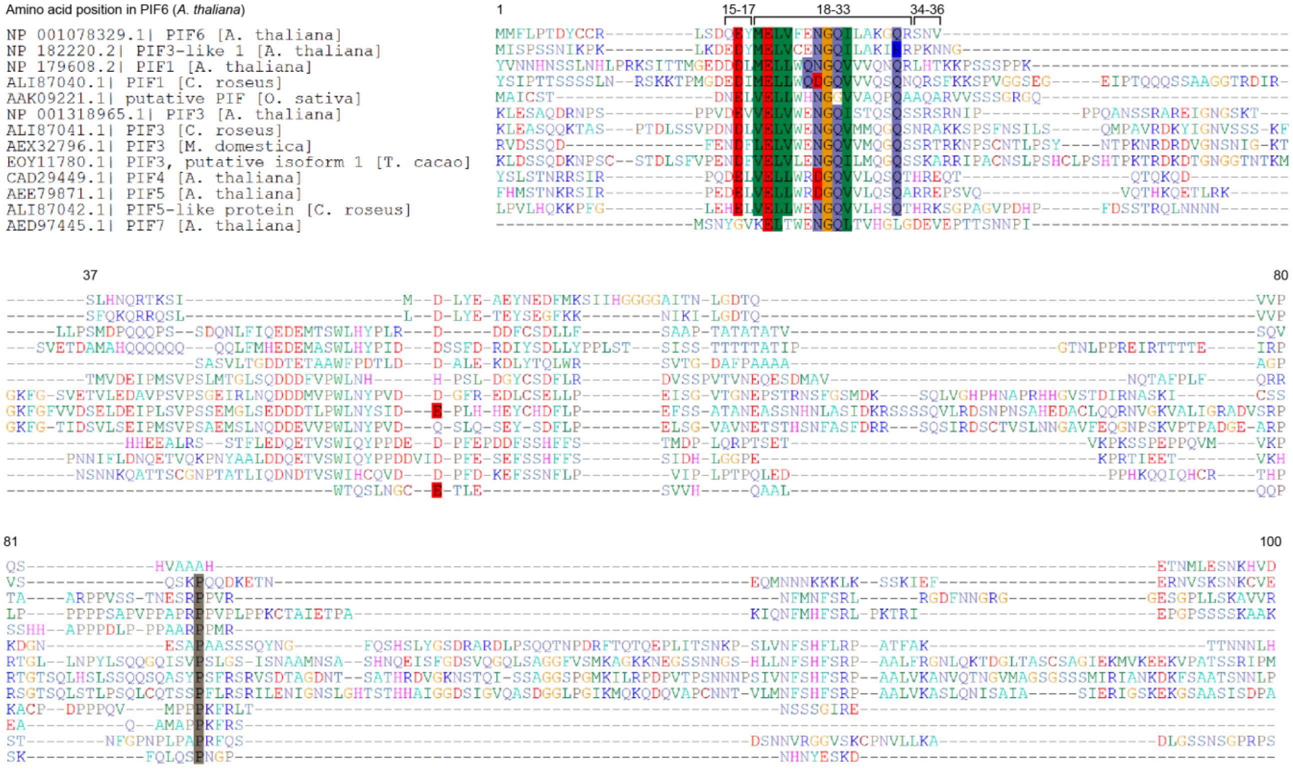

Supplementary Figure 2 | Multiple amino acid sequence alignment of phytochrome interaction factors (PIFs) from different plant species. The depicted PIF sequences (GenBank accession number are shown) were aligned by MAFFT (Katoh and Standley, 2013) (EMBL-EBI) with default parameter settings and displayed using BioEdit (Hall, 1999). Identities and similarities are highlighted as shaded amino acids (90% shade threshold, BLOSUM62 matrix). The amino acid positions are indicated with respect to *A. thaliana* PIF6.

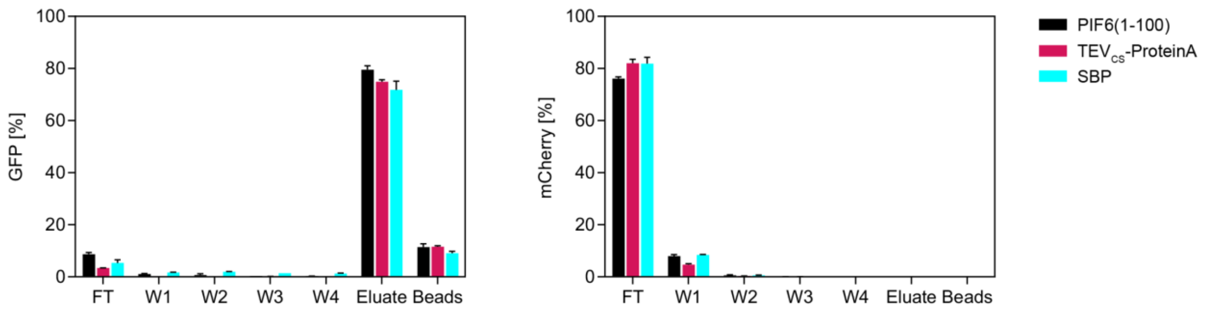

Supplementary Figure 3 | Comparison of the light-controlled affinity purification with established purification methods. HEK-293T cells were transfected with plasmids (see Figure 2A) that encode GFP fused to the depicted different affinity tags and the untagged red fluorescent protein mCherry. During the purification process, fluorescence of GFP and of the background control mCherry was measured and is shown as percentage compared to the ones of the cell lysate. For each replicate, 4×10^6 cells were lysed with 500 µl of lysis buffer and proteins were purified with 50 µl of beads. FT, flow-through; W1-W4, wash 1-4. Data are means ± s.d. (n = 3).

Supplementary Table 5 | Plasmids generated in this study. The following plasmids were used for the cloning: pcDNA3_mζ-SBP (Molnar et al., 2012), pHB111 (Beyer et al., 2018), pLVX-IRES-ZsGreen1 (Clontech, Mountain View, CA), pMH212 (Hörner et al., 2014), pRS315 (Sikorski and Hieter, 1989). The sequences of the used oligonucleotides are depicted in Supplementary Table 6.

Plasmid	Description
pMH501	**5'LTR-P_{CMV}-mCherry-IRES-mGFP_SBP-3'LTR**<br>mCherry was amplified from pMH212 using oligos oMH501 and oMH502; IRES was amplified from pLVX-IRES-ZsGreen1 using oligos oMH418 and oMH419; mGFP was amplified from pHB111 using oligos oMH505 and oMH507; SBP-tag was amplified from pcDNA3_mζ-SBP using oMH508 and oMH509; All four PCR products were Gibson-cloned into EcoRI/MluI digested pLVX-IRES-ZsGreen1.
pMH502	**5'LTR-P_{CMV}-mCherry-IRES-mGFP_TEV_{CS}_proteinA-3'LTR**<br>mCherry was amplified from pMH212 using oMH501 and oMH502; IRES was amplified from pLVX-IRES-ZsGreen1 using oMH418 and oMH419; mGFP was amplified from pHB111 using oligos oMH505 and oMH507; TEV_{CS}_proteinA was amplified from pRS315 using oligos oMH511 and oMH512; All four PCR products were Gibson-cloned into EcoRI/MluI digested pLVX-IRES-ZsGreen1.
pMH503	**5'LTR-P_{CMV}-mCherry-IRES-mGFP_PIF6(1-100)-3'LTR**<br>mCherry was amplified from pMH212 using oMH501 and oMH502; IRES was amplified from pLVX-IRES-ZsGreen1 using oMH418 and oMH419; mGFP_PIF6(1-100) was amplified from pHB111 using oligos oMH505 and oMH513; All three PCR products were Gibson-cloned into EcoRI/MluI digested pLVX-IRES-ZsGreen1.
pMH508	**5'LTR-P_{CMV}-mCherry-IRES-mGFP_PIF6(18-36)-3'LTR**<br>mCherry was amplified from pMH212 using oMH501 and oMH502; IRES was amplified from pLVX-IRES-ZsGreen1 using oMH418 and oMH419; mGFP was amplified from pHB111 using oligos oMH505 and oMH507; PIF6(18-36) was amplified from pHB111 using oligos oMH536 and oMH537. All four PCR products were Gibson-cloned into EcoRI/MluI digested pLVX-IRES-ZsGreen1.
pMH511	**5'LTR-P_{CMV}-ZAP70_PIF6(1-100)-IRES-ZsGreen1-3'LTR**<br>ZAP70 was amplified from pSV10.1 (Arthur Weiss, USA) using oligos oMH531 and oMH532; PIF6(1-100) was amplified from pHB111 using oligos oMH525 and oMH526; Both PCR products were Gibson-cloned into EcoRI/SpeI digested pLVX-IRES-ZsGreen1.
pMH512	**5'LTR-P_{CMV}-mCherry-IRES-mGFP_PIF6(15-33)-3'LTR**<br>This plasmid was cloned as described for pMH508 except that oligos oMH541 and oMH542 were used for amplification of PIF6(15-33) from pHB111.
pMH513	**5'LTR-P_{CMV}-mCherry-IRES-mGFP_PIF6(18-33)-3'LTR**<br>This plasmid was cloned as described for pMH508 except that oligos oMH536 and oMH542 were used for amplification of PIF6(18-33) from pHB111.
pMH516	**5'LTR-P_{CMV}-mCherry-IRES-mGFP_PIF6(15-36)-3'LTR**<br>This plasmid was cloned as described for pMH508 except that oligos oMH541 and oMH537 were used for amplification of PIF6(15-36) from pHB111.
pMH521	**5'LTR-P_{CMV}-ZAP70_PIF6(15-36)-IRES-ZsGreen1-3'LTR**<br>ZAP70 was amplified from pMH511 using oMH531 and oMH573; PIF6(15-36) was amplified from pMH516 using oMH563 and oMH572; Both PCR products were Gibson-cloned into EcoRI/SpeI digested pLVX-IRES-ZsGreen1.

Supplementary Table 6 | Sequences of the oligonucleotides used in this study.

Oligo	Sequence (5'→ 3')
oMH418	ACTAGTTCTAGAGCGGCCGC
oMH419	CATATTATCATCGTGTTTTTCAAAGGAAAACC
oMH501	CACCGACTCTACTAGAGGATCTATTTCCGGTGAATTCGCCACCATGGTGAGCAAGGGCGAGGAGGATAAC
oMH502	AGGGAGAGGGGCGGGATCCGCGGCCGCTCTAGAACTAGTTTACTTGTACAGCTCGTCCATGCCGC
oMH505	GGACGTGGTTTTCCTTTGAAAAACACGATGATAATATGGTGAGCAAGGGCGAGGAGCTG
oMH507	ACCAGCAGAACCTGCGGAGCC
oMH508	CTGTACAAGGGCTCCGCAGGTTCTGCTGGTATGGACGAGAAGACCACCGGCTGGAG
oMH509	CACAAATTTTGTAATCCAGAGGTTGATTGTTCCAGACGCGTTCAGGGCTCCCTCTGGCCCTGGG
oMH511	CTGTACAAGGGCTCCGCAGGTTCTGCTGGTGTCGACGGATCCGAGAATCTTTATTTTCAG
oMH512	CACAAATTTTGTAATCCAGAGGTTGATTGTTCCAGACGCGTCTAAAGAGCCGCGGAATTCGCG
oMH513	CACAAATTTTGTAATCCAGAGGTTGATTGTTCCAGACGCGTTCAGTCAACATGTTTATTGCTTTCCAACATGTTTGTTTC
oMH525	GGCTCCGCAGGTTCTGCTGGT
oMH526	GAGAGGGGCGGGATCCGCGGCCGCTCTAGAACTAGTCAGTCAACATGTTTATTGCTTTCCAACATGTTTGTTTC
oMH531	GACACCGACTCTACTAGAGGATCTATTTCCGGTGAATTCGCCACCATGCCAGACCCCGCGGCGCAC
oMH532	GAACATCATACCAGCAGAACCTGCGGAGCCGGCACAGGCAGCCTCAGCC
oMH536	CTGTACAAGGGCTCCGCAGGTTCTGCTGGTATGGAGCTTGTGTTTGAGAATGGCC
oMH536	CTGTACAAGGGCTCCGCAGGTTCTGCTGGTATGGAGCTTGTGTTTGAGAATGGCC
oMH537	CACAAATTTTGTAATCCAGAGGTTGATTGTTCCAGACGCGTTCAAACGTTGGATCTTTGGCCCTTTGC
oMH537	CACAAATTTTGTAATCCAGAGGTTGATTGTTCCAGACGCGTTCAAACGTTGGATCTTTGGCCCTTTGC
oMH541	CTGTACAAGGGCTCCGCAGGTTCTGCTGGTCAAGAGTATATGGAGCTTGTGTTTGAGAATG
oMH542	CACAAATTTTGTAATCCAGAGGTTGATTGTTCCAGACGCGTTCATCTTTGGCCCTTTGCAAGAATCTGG
oMH563	CAAGAGTATATGGAGCTTGTGTTTGAGAATG
oMH572	GAGAGGGGCGGGATCCGCGGCCGCTCTAGAACTAGTCAAACGTTGGATCTTTGGCCCTTTGC
oMH573	CATTCTCAAACACAAGCTCCATATACTCTTGACCAGCAGAACCTGCGGAGCC

Supplementary Tables 1-4 are available as separate files.

References

Beyer, H.M., Thomas, O.S., Riegel, N., Zurbriggen, M.D., Weber, W., and Hörner, M. (2018). Generic and reversible opto-trapping of biomolecules. *Acta Biomater.* 79:276-282. doi: 10.1016/j.actbio.2018.08.032.

Hall, T.A. (1999). BioEdit: a user-friendly biological sequence alignment editor and analysis program for Windows 95/98/NT. *Nucl. Acids. Symp. Ser.* 41:95-98.

Hörner, M., Kaufmann, B., Cotugno, G., Wiedtke, E., Büning, H., Grimm, D., et al. (2014). A chemical switch for controlling viral infectivity. *Chem. Commun. (Camb.)* 50(71):10319-10322. doi: 10.1039/c4cc03292f.

Katoh, K., and Standley, D.M. (2013). MAFFT multiple sequence alignment software version 7: improvements in performance and usability. *Mol. Biol. Evol.* 30(4):772-780. doi: 10.1093/molbev/mst010.

Molnar, E., Swamy, M., Holzer, M., Beck-Garcia, K., Worch, R., Thiele, C., et al. (2012). Cholesterol and sphingomyelin drive ligand-independent T-cell antigen receptor nanoclustering. *J. Biol. Chem.* 287(51):42664-42674. doi: 10.1074/jbc.M112.386045.

Sikorski, R.S., and Hieter, P. (1989). A system of shuttle vectors and yeast host strains designed for efficient manipulation of DNA in Saccharomyces cerevisiae. *Genetics* 122(1):19-27.
